# Establishment of a Novel Fetal Growth Restriction Model and Development of a Stem-Cell Therapy Using Umbilical Cord-Derived Mesenchymal Stromal Cells

**DOI:** 10.3389/fncel.2020.00212

**Published:** 2020-07-28

**Authors:** Yuma Kitase, Yoshiaki Sato, Sakiko Arai, Atsuto Onoda, Kazuto Ueda, Shoji Go, Haruka Mimatsu, Mahboba Jabary, Toshihiko Suzuki, Miharu Ito, Akiko Saito, Akihiro Hirakawa, Takeo Mukai, Tokiko Nagamura-Inoue, Yoshiyuki Takahashi, Masahiro Tsuji, Masahiro Hayakawa

**Affiliations:** ^1^Division of Neonatology, Center for Maternal-Neonatal Care, Nagoya University Hospital, Nagoya, Japan; ^2^Department of Pediatrics, Nagoya University Graduate School of Medicine, Nagoya, Japan; ^3^Faculty of Pharmaceutical Sciences, Sanyo-Onoda City University, Yamaguchi, Japan; ^4^Clinical Research Center, Division of Biostatistics and Data Science, Medical and Dental University, Tokyo, Japan; ^5^Department of Cell Processing and Transfusion, Institute of Medical Science, University of Tokyo, Tokyo, Japan; ^6^Department of Food and Nutrition, Faculty of Home Economics, Kyoto Women’s University, Kyoto, Japan

**Keywords:** umbilical cord-derived mesenchymal stromal cells, fetal growth restriction, mesenchymal stem cell, neurodevelopment, stem cells

## Abstract

Fetal growth restriction (FGR) is a major complication of prenatal ischemic/hypoxic exposure and affects 5%–10% of pregnancies. It causes various disorders, including neurodevelopmental disabilities due to chronic hypoxia, circulatory failure, and malnutrition *via* the placenta, and there is no established treatment. Therefore, the development of treatments is an urgent task. We aimed to develop a new FGR rat model with a gradual restrictive load of uterus/placental blood flow and to evaluate the treatment effect of the administration of umbilical cord-derived mesenchymal stromal cells (UC-MSCs). To create the FGR rat model, we used ameroid constrictors that had titanium on the outer wall and were composed of C-shaped casein with a notch and center hole inside that gradually narrowed upon absorbing water. The ameroid constrictors were attached to bilateral ovarian/uterine arteries on the 17th day of pregnancy to induce chronic mild ischemia, which led to FGR with over 20% bodyweight reduction. After the intravenous administration of 1 × 10^5^ UC-MSCs, we confirmed a significant improvement in the UC-MSC group in a negative geotaxis test at 1 week after birth and a rotarod treadmill test at 5 months old. In the immunobiological evaluation, the total number of neurons counted *via* the stereological counting method was significantly higher in the UC-MSC group than in the vehicle-treated group. These results indicate that the UC-MSCs exerted a treatment effect for neurological impairment in the FGR rats.

## Introduction

Fetal growth restriction (FGR) causes a wide variety of complications due to prenatal hypoxic/ischemic exposure and is defined as a fetal weight lower than the 10th percentile of the population of the gestation period (Jang et al., 2015). It affects about 20 million infants each year in the world (de Onis et al., 1998; Froen et al., 2004). The causes of FGR include chromosomal abnormalities (Lin and Santolaya-Forgas, 1998), genetic syndromes (Abuzzahab et al., 2003), intrauterine infections (Neerhof, 1995), multiple gestation pregnancies (Blickstein, 2004), and inborn errors of metabolism (Abuzzahab et al., 2003) as fetal factors; maternal factors include clinical diseases (Galan et al., 2001; Infante-Rivard et al., 2002; McCowan et al., 2003), nutritional disorders (Schulz, 2010), and drug use (Lieberman et al., 1994). Among these causes, hypertensive disease of pregnancy (HDP), which develops at a higher rate compared with the other causes, is the main cause of maternal, fetal, and neonatal morbidity and mortality; such fetuses are at an increased risk of FGR, prematurity, and intrauterine death (Kintiraki et al., 2015).

HDP induces chronic ischemia/hypoxia in the uterus (Naderi et al., 2017), to which exposure during the fetal period causes various disorders, including hypoglycemia, feeding intolerance, pulmonary hemorrhage (Sharma et al., 2016a,b), and, in particular, neuro-behavioral abnormalities (Padidela and Bhat, 2003). Neurological developmental disorders include poor exercise quality and developmental retardation (Zuk et al., 2004; Bergvall et al., 2006), and impaired intelligence/cognition, academic problems, mental problems, and obstacles to adaptation in society last for a lifetime (Sharma et al., 2016a). In this way, FGR increases neonatal mortality and morbidity as well as the risk of long-lasting problems in life.

The present study focuses on the neurodevelopmental disorders in FGR associated with chronic mild intrauterine hypoperfusion. Some infants with FGR exhibit abnormalities upon neurological examination, including a lower degree of organization and degraded neurobehavioral profiles, particularly in the orientation and motor domains (Feldman and Eidelman, 2006). Moreover, it is well known that children born with FGR exhibit long-term global cognitive impairment and short-term memory difficulties (Geva et al., 2006). Despite such a diverse range of serious diseases that last for a lifetime, there are few established treatments for neonates with FGR. Aggressive postnatal nutrition, constant rehabilitation, and growth hormone administration for physical growth are often conducted for FGR, but these are just supportive rather than fundamental treatments. Therefore, the development of a novel treatment is an urgent task.

Chronic placental dysfunction is a common cause of FGR, and inadequate blood flow to the placenta during pregnancy results in an inadequate supply of nutrients and oxygen to maintain proper fetal growth (Wixey et al., 2017). Several studies have reported on various FGR models, e.g., exposure to low-concentration oxygen (Morton et al., 2010; Dolinsky et al., 2011), ligation of the uterine artery with silk threads (Ruff et al., 2017), and administration of a synthetic thromboxane A_2_ analog (STA_2_) (Saito et al., 2009). However, considering the blood flow to placentas in FGR, these models do not properly mimic FGR associated with HDP, in which chronic ischemia occurs in the uterus. Therefore, to understand the pathophysiology of FGR more accurately and/or develop a novel therapy for FGR with an animal model, it is necessary to establish a proper model with a chronic decrease of blood flow to the uterus.

In recent years, many reports have demonstrated the effectiveness of stem cell therapy in repairing cells and tissues (Schwarz et al., 1999; Orlic et al., 2001; Wagenaar et al., 2017). Mesenchymal stromal cells (MSCs) have been reported to be a potential source of therapies for various conditions such as stroke, Parkinson’s disease, and myocardial infarction (Schwarz et al., 1999; Chen et al., 2001; Orlic et al., 2001) and can differentiate into various mesodermal tissue cells or neuronal cells (Uccelli et al., 2008). Moreover, MSCs secrete various factors that not only suppress inflammation but also enhance neurogenesis and angiogenesis (Cunningham et al., 2018). Also, MSCs have a low risk of rejection and graft-vs.-host disease (Le Blanc and Ringden, 2005; Götherstrom, 2007). Therefore, we have hypothesized that the ability of MSCs to differentiate into various tissues and anti-inflammatory effects is effective against the tissue injury and persistent inflammation associated with chronic ischemia in FGR. Therefore, MSC-based cell therapy has the potential to be a promising treatment for FGR.

Of the various MSCs, we used umbilical cord-derived MSCs (UC-MSCs) in the present study. The advantages of UC-MSCs are as follows: a noninvasive collection procedure for autologous or allogeneic use, multipotency and low immunogenicity with a good immunosuppressive ability (Girdlestone et al., 2009; Nagamura-Inoue and He, 2014) that is greater in Wharton’s Jelly-derived MSC than in bone marrow-derived mesenchymal stem cell (BM-MSC; Prasanna et al., 2010), easy storage, and few ethical problems for collection and usage. In addition to those advantages, UC-MSCs have demonstrated the ability to accumulate in damaged tissue and differentiate into three different germ layers that induce tissue repair (Nagamura-Inoue and He, 2014). Moreover, UC-MSCs also have a higher proliferative ability than various other MSCs (Hsieh et al., 2010; Sriramulu et al., 2018).

The purposes of the present study are to create a novel FGR rat model with a gradual restrictive load of the uterus/placental blood flow and to evaluate the treatment effects of UC-MSCs administration for the FGR model rats.

## Materials and Methods

### Animals

The animal experiment protocols adopted in the present study were approved by the Institutional Review Board of Nagoya University (Nagoya, Aichi Prefecture, Japan; permit numbers: 27354-2015, 28001-2016, and 29096-2017). The number of animals was kept to the minimum required to achieve statistical significance. Sprague Dawley dams were obtained from Japan SLC Inc. (Shizuoka, Japan). All the rats were allowed free access to food and water and housed in a temperature-controlled room (23°C) under 12:12 h light/dark conditions (9.00 a.m.–9.00 p.m.). We used a total of five dams for the sham group and nine dams for the FGR model i.e., vehicle and UC-MSC groups. Also, we used five dams (three for the sham; fetus *n* = 12 and two for the FGR; fetus *n* = 7) for the measurement of the uterus blood flow. We used 37 pups for the shams group, 18 for the vehicle group, and 15 for the UC-MSC group as negative geotaxis. Then, we separated males and females and used seven males for the sham group, nine for the vehicle group, and seven for the UC-MSC group as well as females for the immunohistological evaluations (eight for the sham group, three for the vehicle group and four for the UC-MSC group). We also used all of the females and males to evaluate the survival rate and safety. The minimal sample size of seven in each group was calculated to achieve an 80% power of testing with an α error rate of 5.0%. This was conducted under the assumption (and based on the preliminary experiments) that the effect size was 1.5 in the rotarod treadmill test, which is a primary endpoint.

### Ameroid Constrictor *in vitro*

**Figure 1:**
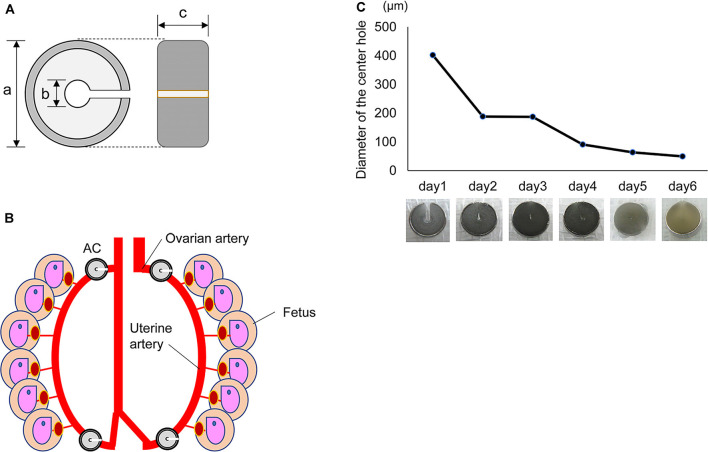
Characters of the Ameroid constrictor. **(A)** Ameroid constrictor. An ameroid constrictor (AC) contains casein inside, has a notch and a hole in the center and is covered with a wide titanium ring. Its size is approximately 3.0 mm in diameter (a); the tool has a center hole of 0.4 mm (b) and a width of about 1.3 mm (c). This casein protein swells slowly when it absorbs water. **(B)** Installation of AC. The figure shows that the left and right ovarian/uterine arteries were sequentially detached from the vein, and an AC was placed in each of the four locations of the artery. **(C)** The change of diameter in AC. The graph shows the diameter of the center hole in an AC in a physiological saline environment of 37°C *in vitro*. The hole shrank to approximately 1/5 its original size in 1 day and to about 1/10 in 3 days. The photograph below shows the change of its shape over time in saline at 37°C.

To induce chronic ischemia, we chose a tool known as an ameroid constrictor (AC; Figure 1A). The AC contains casein inside and is covered with a wide titanium ring approximately 3.0 mm in diameter and 1.3 mm in width, with a notch and a hole in the center of 0.4 mm in diameter (SW-MICE-0.4-SS, Tokyo Instruments Inc., Japan). This casein protein swells slowly when it absorbs water. AC was put into physiological saline at 37°C and the diameter of the center hole was measured every 24 h.

### Surgical Procedure and Implantation of AC

To create an FGR rat model, we induced anesthesia in a rat with 2%–2.5% isoflurane and, then, maintained the anesthesia at around 2% on pregnancy day 17, which is equivalent to 20–25 weeks pregnant in humans (Salmaso et al., 2014), when severe gestation HDP often develops (Li et al., 2020). After confirming that the rat did not respond to painful stimuli, we disinfected its abdomen with alcohol and removed the hair with electric hair clippers, taking care not to damage the teats. Next, we incised the skin and muscular layers about 1.5 cm in the median, somewhat above the second papillary level from the bottom, and confirmed the number of fetuses by pulling out the left and right uteruses. Afterward, we put one uteruses back on one side of the abdominal cavity, while the uteruses on the other side were kept out, covered with gauze, and then humidified and warmed with warm physiological saline and a light bulb. We sequentially peeled the left and right ovarian and uterine arteries from the accompanying veins. Following this, we placed ACs in each of the four arteries (Figure 1B). After placing the ACs, we sewed the muscular and skin layers *via* continuous suturing and closed the incision. Regarding the sham group, we took out the uteruses and counted the number of fetuses. Then, we only waited for 15 min, keeping them humidified and warmed without any treatments, before placing the uteruses back into the abdomen and closing the incision. To reduce stress to the rats, we put nest material in the cage on pregnancy day 15 and made maximum efforts to avoid unnecessary stimulation.

### Blood Flow Change in Uterus With AC *in vivo*

**Figure 2:**
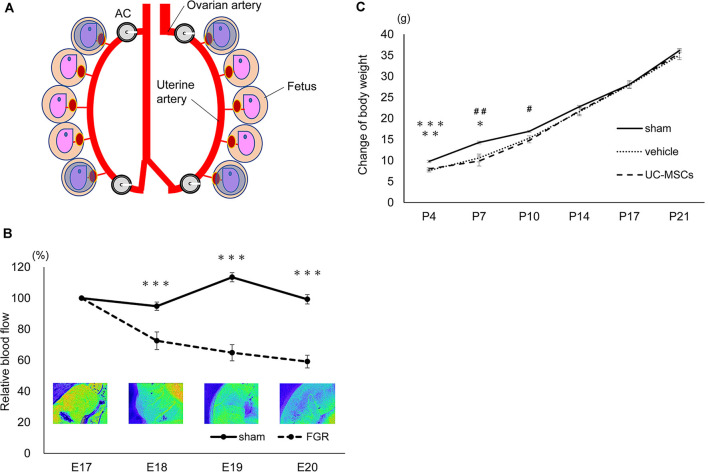
Characters of the fetal growth restriction (FGR) model using the Ameroid constrictor. **(A)** The measurement area. We applied circle ROIs (blue circles) on the inside of the whole uteri and measured the four fetuses: top and bottom of the bilateral uteri before and after AC attachment on embryonic day 17 (E17) as well as E18, 19, and 20. **(B)** The changes in blood flow over time. The measurement of the blood flow every 24 h after the attachment of the AC in the sham group (*n* = 12) and the FGR group (*n* = 7). In the FGR group, the blood flow gradually decreased over time, and on the 20th day of pregnancy, just before birth, the blood flow had decreased to about 60% (sham, *n* = 12; FGR, *n* = 7). ***sham vs. FGR, *p* < 0.001. **(C)** The body weight change of each group. At P4 and P7 days of age, the mean body weight of the sham group was significantly higher than that of the vehicle and the umbilical cord-derived mesenchymal stromal cell (UCMSC) groups. Both the vehicle group and the UC-MSC group subsequently caught up with the sham group, and the significant difference disappeared (sham, *n* = 21; vehicle, *n* = 26; UC-MSCs, *n* = 23). *sham vs. vehicle, *p* < 0.05; ^#^sham vs. UC-MSCs, *p* < 0.05; **sham vs. UC-MSCs, *p* < 0.01; ^##^sham vs. UC-MSCs, *p* < 0.01; ***sham vs. vehicle, *p* < 0.001.

To confirm the changes in blood flow to the uterus derived from the AC attachment, we measured the flow using a laser blood flowmeter ω zone^®^ (OMEGA WAVE, Inc., Tokyo, Japan), which is used to estimate blood perfusion by the speckle contrast (Fredriksson and Larsson, 2017). We defined a circle region of interest (ROI) on the inside of the uterus to cover the whole fetus. We then measured the blood flow in four fetuses in the top and bottom of the bilateral uteri (Figure 2A) in each rat before and after AC attachment on gestational day 17 and on gestational days 18, 19, and 20.

### UC-MSCs

The collection of human umbilical cord tissue and MSC culturing for the present study were approved by the Ethics Committee of the Institute of Medical Science, University of Tokyo, Yamaguchi Hospital, NTT Medical Center Hospital, and Nagoya University Hospital, Japan. The frozen UC-MSCs ready to use were provided by IMSUT CORD in IMSUT. Briefly, the human umbilical cord was collected from babies at the cesarean sections after written informed consent from pregnant mothers. After collecting, the umbilical cord tissue was frozen until use (Shimazu et al., 2015). The frozen-thawed UC tissues were cut into 1- to 2-mm^3^ fragments, covered by Cellamigo^®^ (Tsubakimoto Co., Osaka, Japan) for improved explant isolation (Mori et al., 2015). Tissue fragments were cultured with RM medium (ROHTO Pharmaceutical Co., Ltd., Japan), which is a serum-free culture medium. The fibroblast-like adherent cells that migrated from the tissue fragments were harvested using TrypLE Select (Life Technologies) and these cells were defined as passage 1 cells. The harvested cells were passaged until passage four when the cells were used as UC-MSCs for the experimental analyses. The UC-MSCs were cryopreserved in cryoprotectant, STEM-CELLBANKER^®^ (ZENOAQ Resource Co., Ltd.) and thawed just before use. The cryoprotectant was used as the vehicle in the control group.

### Administration of Treatment and Experimental Design

After AC attachment at pregnancy day 17, we waited for spontaneous delivery. All pups born from rats with ACs were allocated to two equivalent groups at P4 (postnatal day 4), i.e., the UC-MSC group and the vehicle group, based on gender and body weight. Male rats were used for behavioral experiments and females for a histological examination. We administered 1 × 10^5^ UC-MSCs *via* the right jugular vein in the UC-MSC group and only STEM-CELLBANKER^®^ in the vehicle group at P4, which is the same as the preterm of humans and corresponds to the time when the adverse effects on neural development easily develop (Baschat, 2011). The right external jugular vein was exposed under general anesthesia using isoflurane (3.0% for induction and 2.0% for maintenance) on a heating plate set on 37°C and then injection was conducted using a 35-gauge needle. We subsequently performed behavioral experiments at P8–11 and 5 months after birth with the male rats and an immunohistological examination 2 months after birth with the female rats.

### Tissue Preparation and Immunohistochemistry

For the immunohistological evaluation, we used anti-neuronal nuclei (NeuN) antibody (clone A60, EMD Millipore, Burlington, MA, USA), a marker for neurons. The histological and immunohistochemical procedures were performed as previously described (Osato et al., 2010) with minor modifications. Briefly, 2 months after birth, all the female rats were administered pentobarbital sodium intraperitoneally for anesthetization before being sacrificed; then, it was perfused intracardially, first with normal saline and next with 4% paraformaldehyde in phosphate buffer. The rats’ whole brains were collected and immersed overnight in the same paraformaldehyde solution. Subsequently, the brains were transferred to 10% sucrose, stirred gently before sinking, and transferred to 20% sucrose and, finally, to 30%. Next, 40-μm coronal sections were cut throughout the brain every 600 μm.

The sections were blocked with 0.6% H_2_O_2_ in phosphate-buffered saline (PBS), then blocked using 3% normal donkey serum with 0.1% Triton-X100 in PBS for 30 min. These sections were incubated overnight at 4°C with a primary antibody (1:400; mouse anti-NeuN) in 3% donkey serum and PBS. On the second day, the sections were further incubated with a secondary antibody (1:1,000; biotinylated donkey anti-mouse; Jackson ImmunoResearch Laboratories, West Grove, PA, USA) with 3% donkey serum and 0.1% Triton-X100 in PBS for 60 min. After using Avidin-Biotin-Peroxidase (Vectastain Elite ABC Kit; Vector Laboratories, Burlingame, CA, USA) for 60 min, peroxidase detection was performed for 15 min (0.25 mg/ml DAB, 0.01% H_2_O_2_, 0.04% NiCl_2_).

Furthermore, we evaluated the volumes of the cortex, hippocampus, and corpus callosum as well as the number of S100-positive, Iba-1-positive, and double-positive cells for ED-1+/Iba-1+ and CD206+/Iba-1+ using three sections per rat, 20 sections (600 μm) apart, at the hippocampal and basal ganglia level. We applied the same staining protocol to anti-S100 as that applied to anti-NeuN using a primary antibody (rabbit anti-S100; 1:1,000; Dako Cytomation, Glostrup, Denmark) and secondary antibody (1:1,000; biotinylated donkey anti-rabbit; Jackson ImmunoResearch Laboratories, West Grove, PA, USA). We performed triple staining using anti-Iba-1 (1:1,000 Abcam ab5076, Cambridge, UK), anti-ED-1 (1:300; EMD Millipore, Burlington, MA, USA), and anti-CD206 (1:100; Abcam, Cambridge, UK) with overnight incubation at 4°C, followed by incubation with Alexa Fluor goat 546, Alexa Fluor mice 488, and Alexa Fluor rabbit 647 (1:500) antibodies, respectively, for 1 h at room temperature. The sections were then mounted using ProLong Gold Antifade reagent containing DAPI (Thermo Fisher Scientific Inc., Waltham, MA).

### Cell Counting and Volume Measurement

We counted the NeuN-positive cells throughout the hippocampus and cortex *via* unbiased stereological counting techniques as discussed below (Stereo Investigator version 10 stereology software, Micro Bright Field Europe EK, Magdeburg, Germany). After outlining the borders of the hippocampus and the cortex, the computer program overlaid the outlined area with a grid system of counting frames. Cells within these frames as well as those touching two out of four predetermined sides of the frames were counted. The total number of NeuN-positive cells was calculated according to Cavalieri’s principle using the following formula: tN = Σn × P, wherein tN = the total number, Σn = the sum of the number of positive cells in each section, and P = the inverse of the sampling fraction (Osato et al., 2010).

We placed squares (600 × 600 μm) in the bilateral cortex (Supplementary Figure S1) and counted all S100- and Iba-1-positive cells inside each square. The cell counts were expressed as densities. To calculate the Iba-1+/ED-1+ and Iba-1+/CD206+ cells, at least 50 Iba-1-positive cells per animal were analyzed. The ratios of Iba-1+/ED-1+ cells to Iba-1+ cells and Iba-1+/ CD206+ cells to Iba-1+ cells were assessed using a fluorescence microscope (IX83, Olympus Co, Tokyo, Japan) and multiplied by the number of Iba-1-positive cells.

The areas of the cortex and corpus callosum were measured using a Stereo Investigator and the volume of each region was then determined according to the Cavalieri principle: V = ΣA × P, where V = total volume, ΣA = sum of the area measurements, and P = inverse of the sampling fraction.

### Behavioral Tests

#### Negative Geotaxis

For all rats, we performed a negative geotaxis test for four consecutive days, from P8 to P11 (Saito et al., 2009). We placed an anti-slip mat on a 30° slope, facing each rat’s head downward. We measured the time it took each rat to rotate 180° and face their head upward. We scored using zero to five points based on the rotation time: five points for 0–15 s, four points for 15–30 s, three points for 30–45 s, two points for 45–60 s, one point for over 60 s, and zero points for no reaction or falling.

#### Rotarod Treadmill Test

A rotarod treadmill was used to measure the duration of time that each rat could stay on an automatic rotating rod to check their balance, coordination, and stamina. The rats were placed on rotating rods that accelerated from 4 to 40 rpm for 5 min. On the first day, only training (at 4 rpm) was performed to help the animals get used to the environment and rods; the examination was carried out over the following 2 days. We measured twice a day, with a break time of 4 h per day, totaling four observations over 2 days. The amount of time each animal remained on the rod was measured.

### Statistical Analysis

All data were expressed as mean ± standard error. One-way analysis of variance was performed when comparing three groups. *Post hoc* comparisons were made using Tukey’s test. A significant difference was observed at *p* < 0.05. All statistical analyses were performed using JMP 13 (SAS Institute Inc., Cary, NC, USA).

## Results

### Changes in the Inner Hole of an AC *in vitro*

First, we examined how an AC (Figure 1A) changes *in vitro*. We placed an AC into physiological saline at 37°C and measured the diameter of the center hole every 24 h. The hole narrowed to approximately 1/5 of its original size after 24 h and to 1/10 after 3 days. The hole of the AC closed completely within 1 week (Figure 1C).

### Uterine Blood Flow Changes Due to AC Attachment

The blood flow was measured using a laser blood flowmeter after attaching the ACs to the rats on pregnancy day 17 (Figure 1B). The sham group showed almost no change in blood flow. Although no change in blood flow was observed immediately after AC attachment, the flow decreased to 72.5 ± 5.7% after 24 h (sham 94.8 ± 2.7 *p* < 0.001), to 64.8 ± 5.2% after 48 h (sham 113.5 ± 2.9 *p* < 0.001), and to 59.1 ± 4.1% after 72 h (sham 99.3 ± 3.9 *p* < 0.001) compared with the initial blood flow before the attachment of ACs (Figure 2B).

### Survival Rate and Body Weight

The survival rate and weight of the sham group (*n* = 21), vehicle group (*n* = 23) and UC-MSC group (*n* = 26) were compared. To create FGR rats, we performed a surgical operation on 2 rats (dams) for the sham and 6 rats (dams) for the AC attachment. All dams delivered on pregnancy day 21 in both groups.

Regarding the sham group, 21 fetuses in total were confirmed, and all pups were born. No deaths were observed during the observation period thereafter.

In the AC-mounted group, 50 rats (63.3%) of 79 fetuses confirmed upon the operation for AC attachment were born. According to the protocol of the present study, all FGR rats were mixed at P4 and divided into two equivalent groups based on their body weights. Overall, 5 of 24 animals (20.8%) in the vehicle group and 4 out of 26 (15.4%) in the UC-MSC group died by P21. Afterward, all animals in both groups survived to the end of the observation period, i.e., to 310 days of age.

At P4, the mean bodyweight of the sham group was higher (9.8 ± 0.3 g) than that of the vehicle group (7.6 ± 0.3 g; *p* < 0.001) and the UC-MSC group (8.0 ± 0.4 g; *p* < 0.01). The body weight at P7 in the sham group was 14.3 ± 0.3 g vs. 10.6 ± 0.9 g in the vehicle group (*p* < 0.05) and 9.9 ± 1.2 g in the UC-MSC group (*p* < 0.01). Moreover, at P10, the body weight was 16.9 ± 0.2 g in the sham group vs. 15.3 ± 0.6 g in the vehicle group (n.s.) and 14.8 ± 0.6 g in the UC-MSC group (*p* < 0.05). The body weight in both the vehicle and UC-MSC groups subsequently caught up to that of the sham group, and the significant difference among the groups disappeared (Figure 2C).

### Behavioral Tests

#### Negative Geotaxis

To evaluate both the effect of FGR and the treatment effect of UC-MSCs on the maturity of vestibular receptors, central sensory function, and motor function, a negative geotaxis test was performed at P8–11. The time it took the rats to rotate 180°, with the head facing downward on a 30° slope, was measured.

**Figure 3:**
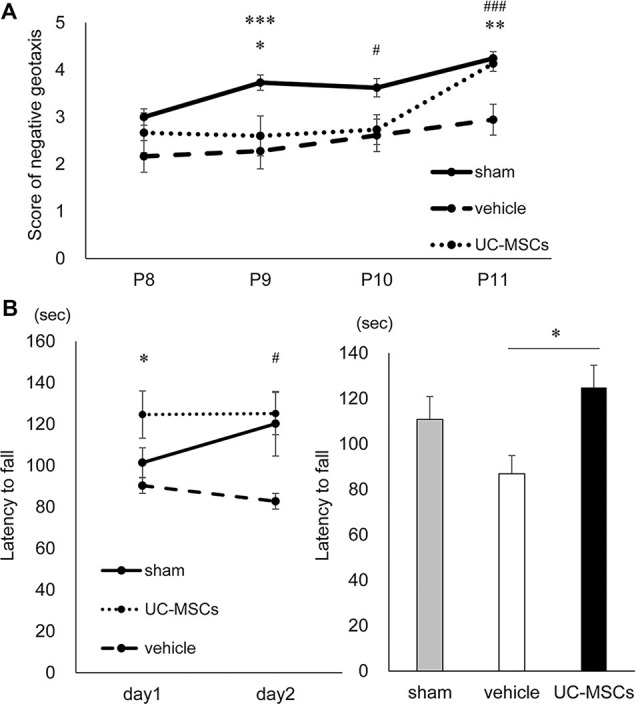
Behavioral tests. **(A)** Negative geotaxis. At postnatal day 9, both the vehicle group and the UC-MSC group were significantly lower scores than the sham group. At postnatal day 10, the vehicle group was significantly lower than the sham group. At postnatal day 11, the vehicle group was significantly lower than the sham group, and the UC-MSC group significantly improved over the vehicle group. Sham group is represented by a solid line, vehicle group by a dashed line and UC-MSC group by a dotted line (sham, *n* = 37; vehicle, *n* = 18; UC-MSCs, *n* = 15). *sham vs. UC-MSCs, *p* < 0.05; ^#^sham vs. vehicle, *p* < 0.05; **UC-MSCs vs. vehicle, *p* < 0.01; ***sham vs. vehicle, *p* < 0.001; ^###^sham vs. vehicle, *p* < 0.001. **(B)** Rotarod treadmill test. The average daily values were calculated. The left bar graph shows a daily average, and the right bar graph does an average of total observations. The duration was shortened in the vehicle group and prolonged in the UC-MSC group at P154-155. Sham group is represented by a solid line, vehicle group by a dashed line and UC-MSC group by a dotted line (sham, *n* = 7; vehicle, *n* = 9; UC-MSCs *n* = 7). *UC-MSCs vs. vehicle, *p* < 0.05; ^#^UC-MSCs vs. vehicle, *p* < 0.05.

No significant difference was observed among the sham group (*n* = 37), vehicle group (*n* = 18), and UC-MSC group (*n* = 15) at P8. The scores in the vehicle group were significantly lower than those in the sham group at P9, P10 and P11 (2.3 ± 0.3 vs. 3.7 ± 0.2 at P9, *p* < 0.001; 2.6 ± 0.3 vs. 3.6 ± 0.2 at P10, *p* < 0.05; 2.9 ± 0.2 vs. 4.2 ± 0.2 at P11, *p* < 0.001, respectively). However, the UC-MSC group’s scores were significantly higher compared with those of the vehicle group at P11 (4.1 ± 0.3 vs. 2.9 ± 0.2, *p* < 0.01, respectively; Figure 3A).

#### Rotarod Treadmill Test

To evaluate balance, exercise coordination, durability, and learning, a rotarod treadmill test were performed at P154–155. The time it took the rats to fall from a forcibly rotating rod was evaluated. The test was conducted twice a day, with a break time of 4 h a day, for a total of four times in total over two consecutive days at 5 months after birth. Compared with the average duration of the sham group, that of the vehicle group (*n* = 9) was shorter (sham vs. vehicle, 110.8 ± 9.6 vs. 86.9 ± 8.5 s, respectively), while that of UC-MSC group (*n* = 7) was significantly longer (UC-MSC vs. vehicle 124.9 ± 9.6 vs. 86.9 ± 8.5 s, *p* < 0.05, respectively; Figure 3B).

### Immunohistological Evaluation

**Figure 4:**
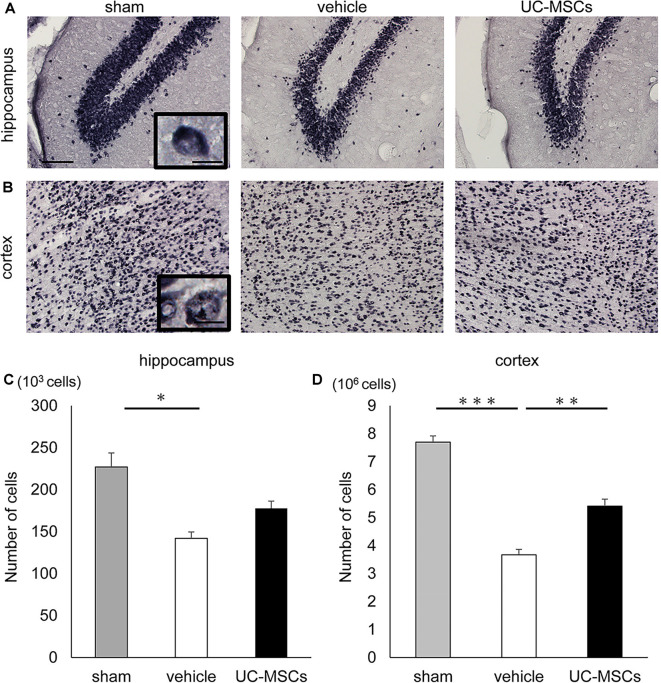
Effects of UC-MSC treatment on neurons in FGR. Representative photomicrographs of the hippocampus **(A)** and part of the cortex **(B)** 2 months after birth. Scale bar = 100 μm. The inset shows a higher magnification view. Scale bar = 10 μm. **(C)** The number of positive cells for NeuN in the hippocampus was significantly lower in the vehicle group than in the sham group, although there was no significant difference between the UC-MSC group and the sham group (sham, *n* = 8; vehicle, *n* = 3; UC-MSCs *n* = 4). *p < 0.05. **(D)** The number of positive cells for NeuN in the cortex was significantly lower in the vehicle group than that in the sham group, and a significant amelioration was observed in the UC-MSC group (sham, *n* = 8; vehicle, *n* = 3; UC-MSCs *n* = 4). **p < 0.01, ***p < 0.001.

An immunohistological evaluation using an anti-NeuN antibody, which recognizes neuron-specific nuclear proteins, was performed to histologically evaluate the impact of FGR and the UC-MSC treatment on the brain. Representative photomicrographs of NeuN-positive cells in the hippocampus (Figure 4A), and cortex (Figure 4B) are shown. The total number of neurons was counted *via* the stereology method. To start, the total number of neurons in the hippocampus was evaluated. The number of positive cells for NeuN in the hippocampus was significantly lower in the vehicle group than in the sham group (141,960 ± 7,626 cells vs. 226,988 ± 16,751 cells, *p* < 0.05), whereas there was no significant difference between the UC-MSC group and the sham group (177,720 ± 8,631 cells vs. 226,988 ± 16,751 cells, n.s.; Figure 4C). Next, the number of positive cells for NeuN in the cerebral cortex was evaluated. As in the hippocampus, the total was significantly smaller in the vehicle group than in the sham group (3,670,185 ± 195,271 cells vs. 7,698,468 ± 221,946 cells, *p* < 0.001), and a significant improvement was observed in the UC-MSC group (3,670,185 ± 195,271 cells vs. 5,408,288 ± 252,595 cells, respectively, *p* < 0.01; Figure 4D).

**Figure 5:**
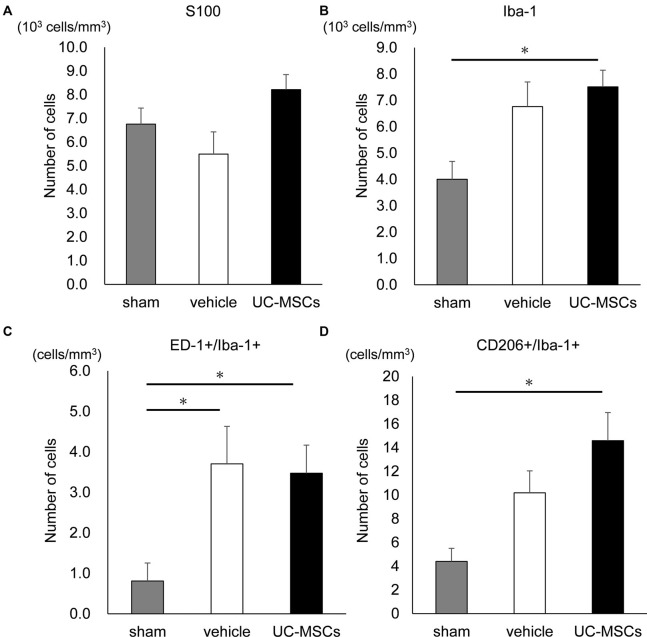
Effects of UC-MSC treatment on astroglia and microglia in FGR. **(A)** There was no significant difference among the three groups in the number of S100 positive cells in the cortex. **(B)** The number of Iba-1 positive cells in the cortex was significantly higher in the UC-MSC group compared to the sham group (sham, *n* = 8; vehicle, *n* = 3; UC-MSCs, *n* = 4). *p < 0.05. The number of ED-1 and Iba-1 double-positive cells **(C)** and CD206 and Iba-1 double-positive cells **(D)** in the cortex with fluorescent immunostaining. The number of ED-1+/Iba-1+ in both the UC-MSCs and the vehicle were significantly higher than that in the sham (sham, *n* = 8; vehicle, *n* = 3; UC-MSCs, *n* = 4). *p < 0.05, whereas that of CD206+/Iba-1+ in the UC-MSCs was significantly higher than that in the sham (sham, *n* = 8; vehicle, *n* = 3; UC-MSCs, *n* = 4). *p < 0.05.

We also evaluated the number of cells that were positive for S100, which is a marker for astroglia that is used to determine the effect on astrogliosis, and Iba-1, which is a marker for pan-microglia to determine the effect on microglia, in the cortex. The numbers of S100 positive cells was not significantly different among the three groups (Figure 5A). However, the number of Iba-1+ cells in the UC-MSC group was significantly higher than that of the sham group (7,511.3 ± 632.1 cells/mm^3^ vs. 4,003.1 ± 678.8 cells/mm^3^, *p* < 0.05; Figure 5B). Therefore, we also evaluated microglia M1 and M2 polarization by counting the number of cells that were double-positive for ED-1+/Ib-1+ and CD206+/Ib-1+, which are markers M1 microglia and M2 microglia, respectively. The numbers of ED-1+/Iba-1+ cells in both the vehicle group and the UC-MSC group were significantly higher compared with the sham group (Figure 5C, vehicle vs. sham, 3.70 ± 0.93 cells/mm^3^ vs. 0.81 ± 0.44 cells/mm^3^ *p* < 0.05; UC-MSC vs. sham, 3.47 ± 0.69 cells/mm^3^ vs. 0.81 ± 0.44 cells/mm^3^, *p* < 0.05), but there was no significant difference between the vehicle group and the UC-MSC group. The number of CD206+/Iba-1+ cells was significantly higher in the UC-MSC group than in the sham group, but there was no significant difference between the vehicle and sham groups (Figure 5D, 14.58 ± 2.37 cells/mm^3^ vs. 4.39 ± 1.11 cells/mm^3^, *p* < 0.05). The volumes of the cortex, hippocampus, and corpus callosum were calculated. We found no significant difference among the sham, vehicle, and UC-MSC groups in either the hippocampus, cortex, or corpus callosum (Supplementary Figure S2).

## Discussion

In the present study, we aimed to create and establish a novel FGR rat model in which the uterine blood flow decreased gradually to investigate the neurological effects of the administration of UC-MSCs for the established FGR model as well as to verify the therapeutic effect of the UC-MSC treatment. To create an appropriate FGR model, we applied a slowly progressive ischemic burden to rats using ACs whose inner central hole gradually narrowed. The rats born with this treatment showed a significant reduction in body weight compared with the sham group, and we confirmed that those who received the ischemic burden in the uterus showed significant abnormalities upon behavioral and immunohistochemical evaluations. Moreover, we confirmed the therapeutic effects of UC-MSCs on the FGR model.

Thus far, a model in which pregnant rats were exposed to hypoxia (Jang et al., 2015), a model in which either a uterine artery was occluded by an atraumatic clamp for 60 min (Kazemi-Darabadi and Akbari, 2020) or a uterine artery and vein were ligated with a thread (Tashima et al., 2001), a model in which STA_2_ was used (Saito et al., 2009), and a model in which microcoils were attached to a uterine and ovarian arteries (Ohshima et al., 2016) have been reported. Herrera et al. (2016) studied a guinea pig model with ACs attaching only to the uterine artery during the second trimester and reported that during pregnancy, the umbilical doppler blood flow decreased, and immediately before birth, the fetuses showed weight loss of approximately 30% and the heart, lungs, liver, and kidneys were significantly reduced in weight, which is similar to that observed in our study; however, they reported no findings regarding neuromotor development. The attaching of ACs to the uterine artery affected the blood flow of each organ and reduced their weight (Herrera et al., 2016). On the contrary, in our FGR model, we attached ACs to not only the uterine but also the ovarian arteries during the third trimester, and the blood flow to the rats’ uteruses was decreased to about 60% before birth *via* ACs whose center hole gradually narrowed. Furthermore, similar to other models (Tashima et al., 2001; Saito et al., 2009; Ohshima et al., 2016), we were able to reproduce a weight loss of over 20%. Although the degree of weight loss was different among litters, the functional impairment induced by chronic intrauterine hypoperfusion was observed in each litter (Supplementary Figure S3). The primary characteristic of the FGR model in the present study was the gradual reduction of blood flow in the uterine and ovarian arteries to the rats’ uteruses.

In the management of HDP, blood flow to the fetus is one of the most important factors determining whether pregnancy can continue (Schwarze et al., 2005). Usually, the blood flow is gradually decreased, and blood flow interruption and sudden blood flow reduction are rare. As such, compared with FGR models using permanent ligations or temporary clamping of uterine arteries, our FGR model more precisely mimicked pathological conditions, as the blood flow to the animals’ uteruses was gradually reduced. The hypoperfused placenta releases inflammatory and anti-angiogenic factors that cause systemic inflammation, vascular dysfunction, high blood pressure, and maternal organ damage (LaMarca et al., 2016). In dogs, bilateral ligation of the uterine ovarian artery and obstruction of the abdominal aorta below the renal artery induced hypertension, proteinuria, and glomerular endotheliosis (Abitbol, 1981). Ligation of the unilateral uterine artery of a baboon also reproduced preeclampsia-like symptoms (Makris et al., 2007). Moreover, the development of FGR in an HDP mother was previously found to be associated with reduced uteroplacental blood flow and placental insufficiency and characterized by decreased oxygen and nutrient delivery to the fetus (Herrera et al., 2014). Although our model was not exactly an HDP model as we did not measure blood pressure and proteinuria in rats with AC in the present study, our model might represent the pathogenesis of HDP.

It is well known that children born with FGR display difficulties in coordination, lateralization, and abundance of associated movements (Leitner et al., 2000). Our model confirmed a significant delay in negative geotaxis as a primitive reflex as in the other model (Saito et al., 2009). Moreover, we revealed impairments of balance, coordination, durability, and learning memory with a rotarod treadmill test in the chronic phase, 5 months after birth. Our FGR rat model displayed similar symptoms as those in children with FGR. However, more experiments are needed to confirm whether our FGR model can display other such symptoms, including cognitive function and behavioral problems, which are partially shown in other FGR models (Tashima et al., 2001; Saito et al., 2009; Pham et al., 2015; Ohshima et al., 2016). In a study that compared the total brain volume in humans using MRI, including a reduction in cortical gray and white matter volume, the small for gestational age (SGA) group showed a total brain volume reduction of almost 6% compared with the non-SGA control group. Thus, it has been reported that cerebral cortex volume is significantly affected by FGR (Østgård et al., 2014). Other studies have reported that SGA causes a decreased white matter volume in the cerebrum and cerebellum, a decreased basal ganglia volume, and a decreased overall cortical surface area (De Bie et al., 2011). In the present study, since both the numbers of neurons in the hippocampus and cortex were significantly reduced, the FGR model has a gray matter injury. To evaluate a white matter injury, we measured the volume of the corpus callosum but we did not find a significant difference among the three groups. Although our FGR model may be unlikely to have a white matter injury, a more detailed evaluation regarding white matter injury is required. Clinical studies have shown that FGR is also associated with diffuse white matter lesions, microglial activation, astrogliosis, and loss of pre-oligodendrocytes followed by a delay in myelination (Olivier et al., 2007), and decreased hippocampal volume (Lodygensky et al., 2008). Other FGR models display white matter (Ohshima et al., 2016) and hippocampal (Ruff et al., 2017) injuries and glial activation (Pham et al., 2015). Further detailed evaluations should be performed to demonstrate how our FGR model histologically mimics FGR in clinical settings.

In the present study, we also observed the therapeutic effects of UC-MSCs on primitive reflexes and motor function. In particular, the FGR model in which UC-MSCs were administered had a significantly large number of neurons. The FGR model delayed neurological maturation and impaired the behavioral deficit, and that administration of UC-MSCs ameliorated the neurological development delay and exerted a significant treatment effect on the deficit, even in the long term, i.e., 5 months after birth. The major mechanisms that may cause FGR brain cell death and injury are presumed to be excitotoxicity, oxidative stress, necrotic and apoptotic degeneration, and neuroinflammation (Rees et al., 2011; Miller et al., 2016). Chronic blood flow disturbances result in decreased oxygen delivery to the brain and decreased glucose and amino acid delivery, potentially affecting immature neurons and glial cells (Rees et al., 2011).

A previous study on the neurological effects of prenatal hypoxia on guinea pig brains showed that brain-derived neurotrophic factor (Bdnf) was significantly lower in the FGR group, and it was also reported that the Bdnf and Tropomyosin receptor kinase B protein levels were reduced in FGR fetuses (Dieni and Rees, 2005; Ke et al., 2011). The UC-MSCs express neurotrophic factors, and it has been reported that the UC-MSC-conditioned medium promotes Schwann cell viability and proliferation through increased levels of nerve growth factor and Bdnf expression due to the paracrine effect of UC-MSC on nerve regeneration (Guo et al., 2015). Our previous study also found that Bdnf and hepatocyte growth factor increased significantly in the serum, cerebrospinal fluid, and brain tissue of mice after intravenous administration of UC-MSCs in a neonatal intraventricular hemorrhage model and that periventricular reactive gliosis, hypomyelination, and periventricular cell death observed after IVH were significantly attenuated (Mukai et al., 2017). Generally, Bdnf is associated with neuronal cell proliferation, survival, and differentiation and is expressed in developing and adult brains (Lee et al., 2002; Pollock et al., 2016). As such, it also relieved hippocampal neuronal loss and promoted neurogenesis in the IVH model (Ko et al., 2018), enhanced proliferation in neuronal populations in the cerebellum and hippocampus (Nonomura et al., 1996; Labelle and Leclerc, 2000; Ko et al., 2018), and ameliorated hypomyelination (Xiao et al., 2010; Peckham et al., 2016). Also, the hepatocyte growth factor is known as a neurotrophic factor of motor, sensory, and parasympathetic neurons (Liu et al., 2010) and affects the development and growth of oligodendrocytes and the proliferation of myelin-forming Schwann cells (Yan and Rivkees, 2002). In a previous study with traumatic brain injury model rats, UC-MSC administration up-regulated neurotrophic factors, such as Bdnf and hepatocyte growth factor, and improved the neurological severity (Qi et al., 2018). In the present study, neurotrophic factors secreted by the UC-MSCs likely suppressed the neuroinflammation induced by chronic ischemia. The results of perinatal brain injury heavily involve microglia; proinflammatory M1 microglia first respond to stimuli such as hypoxic ischemia and infection and then involve the production of inflammatory cytokines that ultimately exacerbate brain injury. After the pro-inflammatory phase, the anti-inflammatory M2 microglia initiates the production of anti-inflammatory molecules that promote tissue repair and nerve regeneration (Hagberg et al., 2015). Exosomes derived from Human Wharton’s jelly mesenchymal stem cells reduce microglia-mediated neuroinflammation with perinatal brain injury (Thomi et al., 2019). In the present study, there was no significant effect of UC-MSCs on M1 microglia. However, the number of M2 microglia was significantly higher in the administration of UC-MSCs than the sham. One possible mechanism for the improvement of the behaviors in the present study might be *via* M2 microglia activated by UC-MSCs. The inflammation and apoptosis in the acute and subacute phases could not be evaluated directly in the present study, but the decrease in the number of neurons was improved, and the number of M2 microglia, which result in an anti-inflammatory effect, was increased by UC-MSC administration. We speculated that UC-MSC treatment resulted in a reduction in cell death and/or an ameliorated suppressed neurogenesis *via* suppression of inflammation, which led to behavioral improvement. In the subsequent studies, we will evaluate the effect on inflammation and apoptosis in detail.

There are several limitations. The present study demonstrated the efficacy of xenograft administration of human UC-MSCs to rats. Before pursuing clinical application, it will be necessary to confirm the safety and efficacy of the human cells used in the clinical trial in a non-clinical study with an animal model. On the other hand, when we administer human cells to rats, we must consider the effects of xenografting. Although the effect of xenograft administration may be small as UC-MSCs have immunomodulatory properties (Nagamura-Inoue and He, 2014), subsequent studies should evaluate the treatment effect using rat derived UC-MSCs.

Regarding the change of the inner diameter of ACs, we only used *in vitro* and not *in vivo* confirmation and this was another drawback of the study. A mouse model of subcortical infarcts using AC (inner diameter: 0.5 mm) reported that the center hole was not completely occluded a week after implantation *in vivo* (Hattori et al., 2015). Although we used a different AC size (inner diameter: 0.4 mm), the AC *in vivo* could be occluded slower than *in vitro*. However, the actual blood flow is more important than the change of AC, and we think the change of AC *in vitro* in the present study may be a reference value.

We administered UC-MSCs on day 4 but were not able to evaluate other treatment times. The third trimester is critical timing for glial cell proliferation and dendritic branching of synapse formation (de Graaf-Peters and Hadders-Algra, 2006). Since FGR injury developed in utero, it may be presumed that earlier timing after birth could be better, but one of the reasons that we selected P4 for the administration of the cells in the present study was due to technical limitations as the size of the pups was too small for us to intravenously administer. We need to further examine the optical number of cells, number of administrations, and timing for UC-MSC treatment.

Also, concerning the immunohistological evaluation, although there were statistically significant differences in some analyses with a limited number of samples, a confirmatory evaluation with a larger number of samples should be performed in the subsequent study.

In conclusion, we established a novel FGR rat model that mimics FGR associated with chronic mild intrauterine hypoperfusion. Also, the intravenous administration of UC-MSCs led to a significant amelioration of the reduced number of neurons and impaired behaviors induced by FGR.

## Data Availability Statement

The datasets generated for this study are available on request to the corresponding author.

## Ethics Statement

The animal study was reviewed and approved by the Institutional Review Board of Nagoya University.

## Author Contributions

YK, YS, MT, MI, AS, YT, and MH designed the study. YK, AO, and MJ made FGR model rats. YK, SA, SG, HM, and TS conducted the experiments with immunohistochemistry, behavioral tests. KU, TM, and TN-I conducted UC-MSCs cell culture. YK and YS analyzed the data. YK, HM, and AH performed statistical analyses. YK and YS wrote the manuscript draft. MI, AS, YT, and HM critically revised the manuscript. All authors contributed to manuscript revision, read and approved the submitted version.

## Conflict of Interest

The authors declare that the research was conducted in the absence of any commercial or financial relationships that could be construed as a potential conflict of interest.

The handling Editor declared a past collaboration with one of the authors YS.
