# Supplementary material for: Establishment of a Novel Fetal Growth Restriction Model and Development of a Stem-Cell Therapy Using Umbilical Cord-Derived Mesenchymal Stromal Cells
Source: Front Cell Neurosci. 2020 Jul 28;14:212. doi: 10.3389/fncel.2020.00212 (PMC7401876; doi:10.3389/fncel.2020.00212)
Supplement: Supplementary file 1 [file Table_1.DOCX]

**Supplementary material**

Establishment of a novel fetal growth restriction model and development of a stem-cell therapy using umbilical cord-derived mesenchymal stromal cells

Yuma Kitase^1,2^, Yoshiaki Sato^1*^, Sakiko Arai^1^, Atsuto Onoda^1^, Kazuto Ueda^1^, Shoji Go^1^, Haruka Mimatsu^1,2^, Mahboba Jabary^1,2^, Toshihiko Suzuki^1^, Miharu Ito^1^, Akiko Saito^1^, Akihiro Hirakawa^3^, Takeo Mukai^4^, Tokiko Nagamura-Inoue^4^, Yoshiyuki Takahashi^2^, Masahiro Tsuji^5^, Masahiro Hayakawa^1^

1. Division of Neonatology, Center for Maternal-Neonatal Care, Nagoya University Hospital, Nagoya, Japan
2. Department of Pediatrics, Nagoya University Graduate School of Medicine, Nagoya, Japan
3. Division of Biostatistics and Data Science, Clinical Research Center, Tokyo Medical and Dental University, Japan
4. Department of Cell Processing and Transfusion, the Institute of Medical Science, the University of Tokyo, Tokyo, Japan
5. Department of Food and Nutrition, Faculty of Home Economics, Kyoto Women's University, Kyoto, Japan

# Supplemental Materials and Methods

**Supplemental Figures**

Supplemental Figure S1


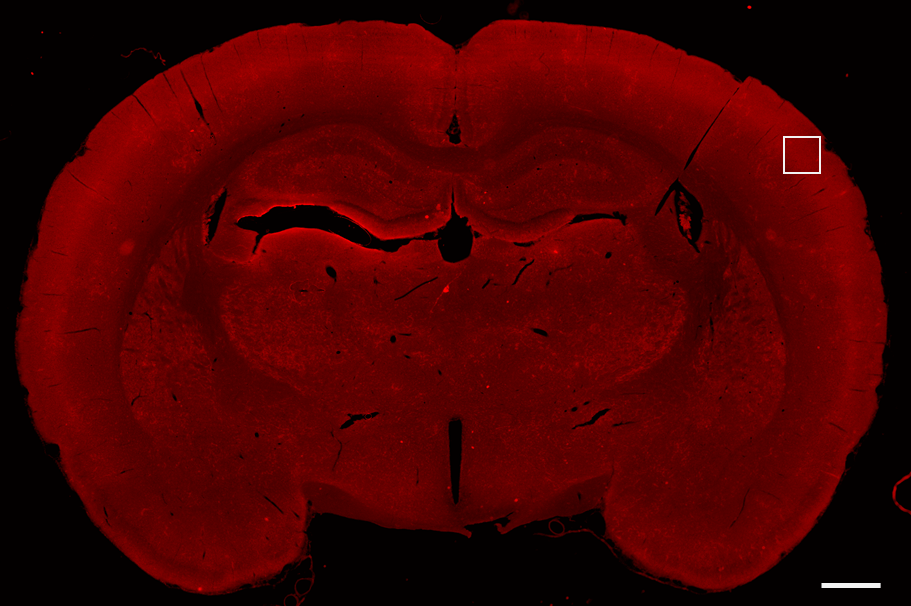


S1: Cell-counting area in the cortex. A square (600 × 600 µm) was placed on the cortex. Bar = 1,000 µm

Supplemental Figure S2


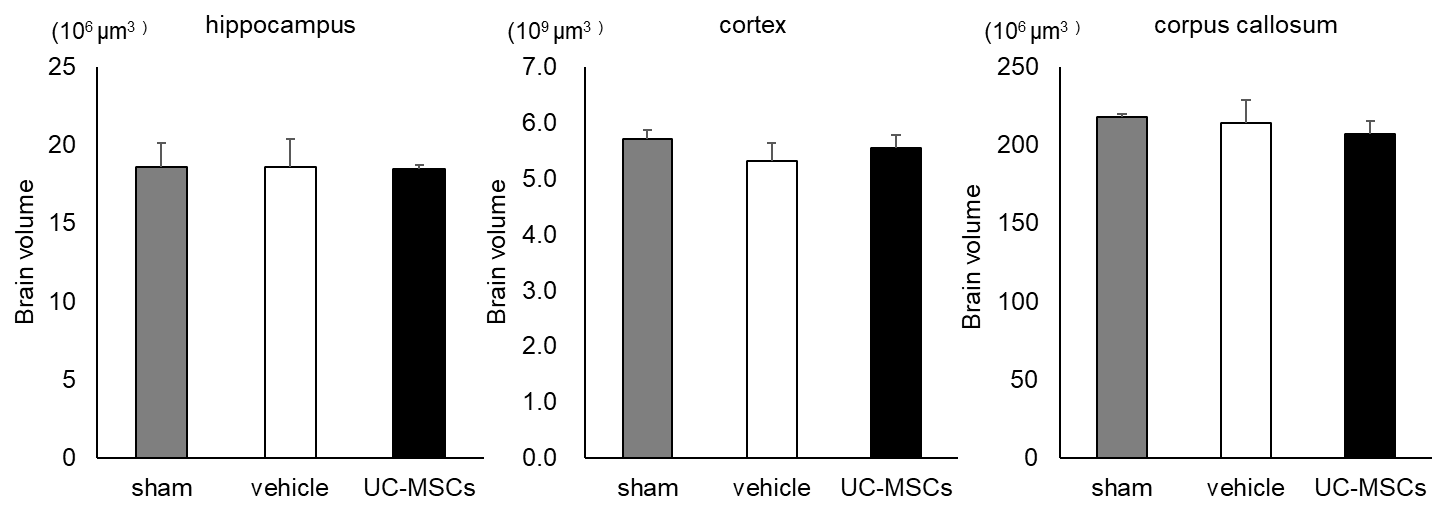


S2: Each volume of the hippocampus, cortex, and corpus callosum was calculated using Stereo Investigator 2 months after birth. Neither the FGR nor the UC-MSCs treatment impacted tissue loss (sham, n = 8; vehicle, n = 3; UC-MSCs, n = 4).

Supplemental Figure S3


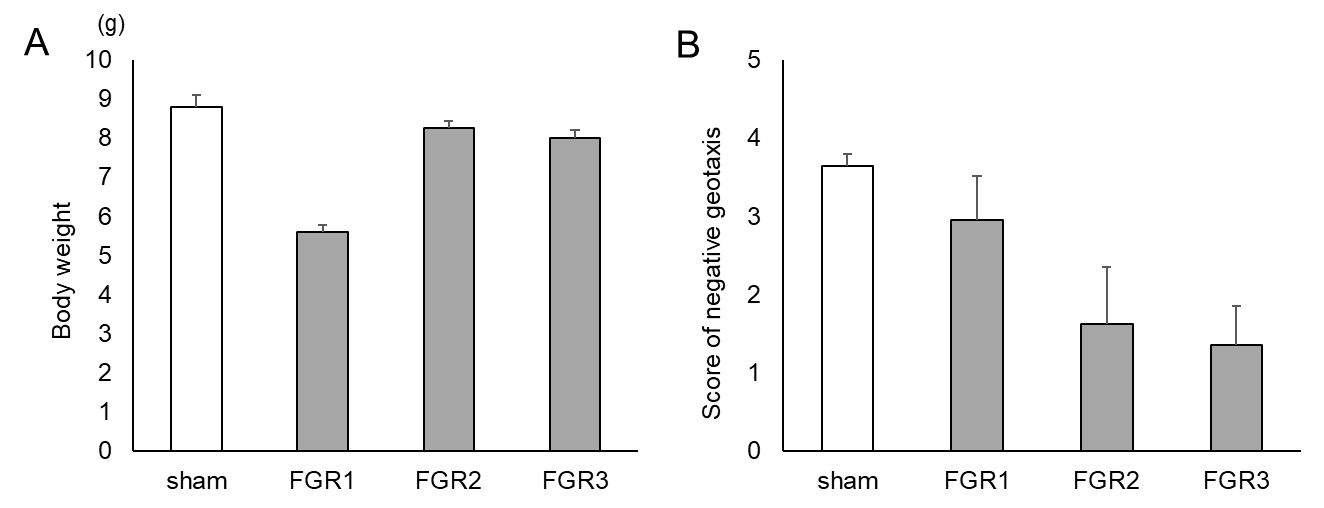


A: Comparison of body weight at P4 between sham and each FGR litter. In each FGR litter, weight loss was recognized, compared with sham (FGR1: n = 11, FGR2: n = 12, FGR3: n = 10).

B: The negative geotaxis score at P9 in each FGR litter (only the vehicle group). The functional impairment induced by chronic intrauterine hypoperfusion was observed in each litter (FGR1: n = 7, FGR2: n = 5, FGR3: n = 6).
